# “Is Romanism Overwhelming Us?”

**Published:** 1888-02

**Authors:** 


### "IS ROMANISM OVERWHELMING US?"

This is a query answered in a decidedly negative way by Rev. Dr. Charles S. Pomeroy, a Presbyterian, of Cleveland, O. He has been looking up the subject carefully and publishing the results in a church magazine. During the last ten years, the increase of Romanism has not been what it was formerly. Though the vast proportion of our immigration has been Roman Catholic, and this element with its descendants may include nearly half our entire population, the latest statistics give less than seven millions of Roman Catholics, including men, women and children. If they had merely held their own, says Dr. Pomeroy, they would have numbered 22,000,000 to-day, instead of less than seven. Dr. Pomeroy makes a very encouraging comparison by giving clerical statistics as follows: "From 1850 to 1880, Romish priests increased 5100; but, meanwhile, Presbyterian ordained ministers increased 4256, Baptists, 11,428, and Methodists, 15,430, to say nothing of the large growth in other denominations. The aggregate increase was 44,315 evangelical ministers, to match about 5,000 priests. Then, estimating the evangelical population by adding only two for each enrolled communicant, it has grown within the past ten years alone, more than six times as fast as the Romish population, and the proportion seems to be rising every year.

